# Managing workflow executions with WESkit

**DOI:** 10.1093/bioinformatics/btag091

**Published:** 2026-02-24

**Authors:** Valentin Schneider-Lunitz, Philip R Kensche, Landfried Kraatz, Philipp Strubel, Stefan Borufka, Gurudeep Parala, Alexander Kanitz, Roland Eils, Ivo Buchhalter, Sven O Twardziok

**Affiliations:** Berlin Institute of Health at Charité—Universitätsmedizin Berlin, Center of Digital Health, Berlin 10117, Germany; Division Omics IT and Data Management Core Facility (ODCF), German Cancer Research Center (DKFZ) Heidelberg, Im Neuenheimer Feld 280, 69120 Heidelberg, Germany; Berlin Institute of Health at Charité—Universitätsmedizin Berlin, Center of Digital Health, Berlin 10117, Germany; Berlin Institute of Health at Charité—Universitätsmedizin Berlin, Center of Digital Health, Berlin 10117, Germany; Division Omics IT and Data Management Core Facility (ODCF), German Cancer Research Center (DKFZ) Heidelberg, Im Neuenheimer Feld 280, 69120 Heidelberg, Germany; Division Omics IT and Data Management Core Facility (ODCF), German Cancer Research Center (DKFZ) Heidelberg, Im Neuenheimer Feld 280, 69120 Heidelberg, Germany; BotCraft GmbH, München 80807, Germany; Biozentrum, University of Basel, Basel 4056, Switzerland; Swiss Institute of Bioinformatics, Amphipôle, Quartier UNIL-Sorge, Lausanne 1015, Switzerland; Berlin Institute of Health at Charité—Universitätsmedizin Berlin, Center of Digital Health, Berlin 10117, Germany; German Center for Mental Health (DZPG), Partner Site Berlin/Potsdam, Germany; German Center for Child and Adolescent Health (DZKJ), Partner Site Berlin, Germany; Division Omics IT and Data Management Core Facility (ODCF), German Cancer Research Center (DKFZ) Heidelberg, Im Neuenheimer Feld 280, 69120 Heidelberg, Germany; Berlin Institute of Health at Charité—Universitätsmedizin Berlin, Center of Digital Health, Berlin 10117, Germany; German Center for Mental Health (DZPG), Partner Site Berlin/Potsdam, Germany; German Center for Child and Adolescent Health (DZKJ), Partner Site Berlin, Germany

## Abstract

**Summary:**

In biomedical research, managing computational workflows across numerous projects—with varying parameters, tools, and environments—creates major challenges in scalability, reproducibility, and collaboration. Here, we present WESkit, an implementation of the Global Alliance for Genomics and Health (GA4GH) Workflow Execution Service (WES) interface, designed to streamline the execution, monitoring, and documentation of data processing workflows. It addresses the complexities involved in managing numerous executions with varying parameters across diverse research projects. Supporting both Snakemake and Nextflow, the system enables consistent automation and centralized monitoring, which benefits research groups aiming for long-term reproducibility and scalable collaboration. Its suitability for larger teams and service units is further enhanced by seamless integration into cloud environments, contributing to the GA4GH cloud framework.

**Availability and implementation:**

The software WESkit is available under MIT license at the GitLab repository (https://gitlab.com/one-touch-pipeline/weskit). The WESkit main repository is archived at Software Heritage (https://archive.softwareheritage.org/browse/origin/directory/?origin_url=https://gitlab.com/one-touch-pipeline/weskit/api.git) and can be found using “one-touch-pipeline/weskit” term in the search section.

## 1 Introduction

The rapid development of novel technologies in the field of biological and medical research has resulted in the generation of overwhelming amounts of data. Examples from the field of health research include genomics, transcriptomics and proteomics data or electronic health records (Cirillo and Valencia 2019). As the volume of data increases, so do the demands on research data management to maintain high data quality, ensuring reproducibility and enabling reusability of data. To this aim, in the field of research data management, following the FAIR (Findable, Accessible, Interoperable, Reusable) criteria is established (Wilkinson *et al.* 2016). The FAIR criteria emphasize the importance of transparency and traceability in the entire data processing chain, from sampling to data analysis and sharing of data. These activities can be well represented as different phases of the data life cycle, with each phase having its own challenges and requiring specific technical as well as organizational solutions (Fatima *et al.* 2023).

The data processing phase typically involves the use of various tools to transform, annotate and integrate raw data in preparation for the subsequent analysis phase (Wing 2019). In bioinformatics communities, this process is often orchestrated through workflow management systems (WfMS) such as Snakemake (Molder *et al.* 2021) and Nextflow (Di Tommaso *et al.* 2017), which enable the efficient execution of complex data processing pipelines. Such workflow systems are also widely used across diverse research domains to support individual researchers and core facilities in execution of selected workflows (Ahmed *et al.* 2021). Workflows can be shared and reused through community driven repositories such as WorkflowHub (Gustafsson *et al.* 2025) or nf-core (Ewels *et al.* 2020). Furthermore, the outcomes of workflow executions can be represented in an interoperable manner by using RO-Crates, which records the output data generated by applying a specific workflow on a defined dataset (Leo *et al.* 2024).

However, challenges remain in the automation and management of large numbers of workflow executions with different parameters and input files. Additionally, the use of different workflow management systems and setting up software environments is challenging especially for experimental scientists. To address these challenges, the Global Alliance for Genomics and Health (GA4GH) has defined the Workflow Execution Service (WES) Application Programming Interface (API) specification for the standardized execution of workflows (Rehm *et al.* 2021). The GA4GH WES API provides a unified way for executing workflows using different workflow management systems.

WESkit implements the GA4GH WES API and handles execution, monitoring and documentation of workflow runs. Initially conceived in the context of the collaboration between Charité Universitätsmedizin Berlin and the Deutsches Krebsforschungszentrum (DKFZ) as part of the German Network for Bioinformatics Infrastructure (de.NBI), WESkit was designed to function as a backend for the data management system One-Touch-Pipeline (OTP) (Reisinger *et al.* 2017). WESkit is engineered to manage both Snakemake and Nextflow workflow executions either on-premises or on remote computing infrastructure. Its scalability enables concurrent submission of numerous jobs, while its design enables addition of workflow management systems, which facilitates long-term maintainability of WESkit as a backend for OTP and independent WES implementation.

## 2 Methods

WESkit is a web service developed using Flask and uWSGI in Python, designed to implement the GA4GH Workflow Execution Service (WES) API specification (version 1.1.0). The development of WESkit follows an object-oriented design, which facilitates modularity and extensibility. Variable features, such as workflow engines, container systems, database access, and cluster systems, are encapsulated in internal APIs. Components such as the database can be easily replaced. The service handles requests asynchronously by using Celery workers (https://github.com/celery/celery, v5.3.4), which manage the submission of workflow runs to the compute infrastructure or querying the status of jobs. Workflow run metadata and job states are persistently stored in a MongoDB database providing unified documentation of workflow executions. User authentication relies on OpenID Connect (OIDC), which was integrated into WESkit during the BioHackathon Europe 2021 (Kensche *et al.* 2023) (Supplementary Data, Fig. 1, available as supplementary data at *Bioinformatics* online). Here, the user authenticates at the client side and sends the job request with a JWT to the WESkit server. The jobs are carried out by the server on behalf of the authenticated user. Files generated as job results are generally not managed by WESkit but rather by different processes, e.g. by a designated data manager. In addition to the WES specification, WESkit also implements a custom API endpoint that provides information about available workflows and the required configurations.

WESkit can be deployed either with Docker Compose or on a Kubernetes cluster using Helm charts, and it supports the execution of workflows either in High Performance Computing (HPC) or Kubernetes clusters. Both deployment options share a consistent configuration structure, although they differ in system details. To improve performance, providers can scale workflow processing by adding more API instances and workers. Thanks to WESkit’s architecture, this scaling is linear (Supplementary Data, Fig. 2, available as supplementary data at *Bioinformatics*  online, Table 1, available as supplementary data at *Bioinformatics* online). During the initial configuration, the working directory for workflow execution and workflow engine parameters must be defined. WESkit is engineered to submit requests to cloud or remote compute infrastructures, including the HTC scheduling systems LSF and SLURM and the execution within container orchestrations engines.

## 3 Use case

Building on the concept of a standardized WES interface, WESkit was originally developed to replace OTP’s own direct submission and monitoring of workflows, which require intimate knowledge of different WfMSs as well as of the cluster backend. As a backend system, WESkit is designed to execute workflows of different types on a cluster system in the numbers relevant for the DKFZ. In the time range from October 2024 to 2025, about 20 000 cancer and research samples were processed in a total of 660 000 workflow runs. Over time, these workflows have also been applied to process pan-cancer data (Aaltonen *et al.* 2020). Although not all workflows have been migrated to Snakemake or Nextflow yet, the integration of OTP and WESkit is ongoing and the WESkit-based submission of the first Nextflow-based FASTQC workflow is in a preproduction state. Sequenced samples will be analyzed using workflows, with reproducibility ensured through containerization (Supplementary Data, Fig. 3, available as supplementary data at *Bioinformatics* online). In general, the use of WESkit can support individual researchers, as well as data management teams and core facilities in the management and automation of multiple workflows.

To improve the user experience, a weskit-client tool that provides easy access to the functionalities of WESkit via a command line terminal was implemented. The tool features the initialization and configuration of workflow executions. When initializing a new workflow run, the weskit-client creates an empty *config.yaml* file. The user configures the workflow execution according to the requirements, e.g. by referencing raw data or through parameterization using the *config.yaml* file. After configuration, the user can start the execution via weskit-client. With the client, scientists can inquire WESkit about the execution status and results of workflow runs. A tutorial for deploying WESkit and using the client is available in the Supplementary Data, available as supplementary data at *Bioinformatics* online.

## 4 Discussion

WESkit supports researchers in data management by providing a user-friendly and reliable environment for the execution of workflows. Its consistent interface enables automation, monitoring, and simplifies integration with additional data management tools through the WES API. Using WESkit is particularly beneficial when specific workflows need to be established on a long-term basis within a research group or organization and these workflows are frequently used for different datasets. However, implementation requires technical expertise for initial setup, including the configuration of the deployment, setting up the connection to the computing environment and integrating workflows. There might also be demand for ongoing maintenance and regular updates of integrated workflows.

By supporting two widely used workflow management systems, WESkit actively advances the application of the FAIR principles in biomedical data management. WfMS contribute to application of FAIR principles by enabling container integration, supporting workflow registries, and by providing detailed execution logs (Wilkinson *et al.* 2025). The standardization of workflow submissions, implemented and thus promoted by WESkit, improves FAIRness, because it streamlines workflow execution and simplifies automation by reducing technical barriers. Its adoption fosters interoperability by ensuring compatibility with diverse data management tools and seamless integration into existing infrastructures. To support secure and controlled access, WESkit and the weskit-client tool incorporate authentication via OIDC-compliant identity providers, such as Life-Science Login enabling federated identity management across cloud environments (Harrow *et al.* 2021). Finally, WESkit enhances data reusability by generating unified documentation for each workflow run, including input files, configurations, and parameter settings. This structured metadata supports reproducibility, provenance tracking, and informed reuse of both workflows and data. Additionally, RO-Crate support will be anticipated to extend WfMS created RO-Crates by WESkit execution logs.

Other approaches and software exist to implement the GA4GH WES API or to provide unified execution of different workflow engines. Notably, the service Sapporo implements the WES API and aims to offer a unified execution for various workflow systems, providing scientists with easier access to different workflow systems (Suetake *et al.* 2024). Thereby, Sapporo offers great flexibility for using different workflow systems, but it does not implement HTC or Kubernetes support natively, although it can leverage these technologies through the underlying implementations of supported workflow engines. The software WfExS supports the unified execution of workflows without providing a REST service implementing the GA4GH WES API (https://github.com/inab/WfExS-backend). WfExS focuses on the unified documentation of workflow execution by implementing RO-Crates natively. In contrast, the focus of WESkit lies on the automation and stable execution of workflows on HTC and cloud systems, making it suitable for larger teams and service units rather than individual scientists. For a detailed comparison see Supplementary Data, Table 2, available as supplementary data at *Bioinformatics* online.

WESkit is particularly suited for the implementation of cloud environments. Besides the WES API, GA4GH is developing the Task Execution Service (TES), Data Repository Service (DRS), and Tool Repository Service (TRS) interfaces within its Cloud Work Stream. For instance, the TES API is used for executing individual tools within federated networks (Kanitz *et al.* 2024) and is supported by Snakemake (Molder *et al.* 2021). Via the TES-client support of WfMSs, WESkit can also be operated as an important component of a GA4GH cloud framework using TES as compute backend. Furthermore, during the BioHackathon Europe 2021 (Kensche *et al.* 2023) a prototype TRS-client support was implemented in WESkit. Although native TRS support is not yet fully implemented, future development will focus on enhancing WESkit’s interoperability with GA4GH Cloud Work Stream standards. Currently, it is up to the respective WESkit administrator of a WESkit instance to make workflows from a TRS repository (e.g. WorkflowHub) available to users. Planned enhancements include also partial TRS compliance for listing available workflows installed in WESkit. As a practical cloud use case, the de. NBI cloud, a federated research cloud in Germany, is exploring WESkit’s role in provisioning of cloud-based Secure Processing Environments (SPEs), enabling controlled execution of validated workflows on sensitive datasets (Braun *et al.* 2025).

The DKFZ and Charité Universitätsmedizin Berlin use WESkit for executing workflows via the data management tool OTP. The OTP system is developed, maintained, and used at DKFZ and the Charité to manage and process substantial amounts of molecular and clinical data generated internally and by cooperating partners. For internal use, WESkit has proven to be a stable and flexible tool for executing and monitoring workflows. Further development of WESkit is driven by a joint team from both institutions and is supported as a long-term service by the German Bioinformatics Network de. NBI. As an open-source project, the further development of WESkit is also open to the community.

### Acknowledgements

This work was supported by the de.NBI Cloud within the German Network for Bioinformatics Infrastructure (de.NBI) and ELIXIR-DE (Forschungszentrum Jülich and W-de.NBI-001, W-de.NBI-004, W-de.NBI-008, W-de.NBI-010, W-de.NBI-013, W-de.NBI-014, W-de.NBI-016, W-de.NBI-022).

## Supplementary Material

btag091_Supplementary_Data

## Data Availability

There are no new data associated with this article.
