# Supplementary material for: Managing workflow executions with WESkit
Source: Bioinformatics. 2026 Feb 24;42(3):btag091. doi: 10.1093/bioinformatics/btag091 (PMC13032820; doi:10.1093/bioinformatics/btag091)
Supplement: btag091_Supplementary_Data [file btag091_supplementary_data.docx]

Managing Workflow Executions with WESkit

Valentin Schneider-Lunitz^1*^, Philip R. Kensche^2*^, Landfried Kraatz^1*^, Philipp Strubel^1^, Stefan Borufka^2^, Gurudeep Parala^2,3^, Alexander Kanitz^4,5^, Roland Eils^1,6,7^, Ivo Buchhalter^2✉^, Sven O. Twardziok^1,6,7✉^

**Supplementary Data**

1. Performance Benchmark
2. Workflow execution services
3. User Tutorial
4. Supplementary Figures and Tables

# Performance benchmark

The benchmarks were conducted using virtual machines hosted on the de.NBI cloud infrastructure, leveraging OpenStack virtualization technology. To benchmark the scalability of the submission frontend and worker backend, two separate virtual machines were utilized: one dedicated specifically to job submissions, and the other configured for WESkit deployment. The WESkit deployment VM was provisioned with 64 virtual cores (Intel Broadwell, 1.9 GHz), 500 GB of RAM, and ran Ubuntu 24.04.1 as the operating system. For each benchmark setup, WESkit was freshly deployed (commit *c84cfb5a*), with one single threaded API container using 3 uWSGI workers. The workflows were executed with varying numbers of Celery workers, each running in a separate container with 20 threads available, to systematically evaluate scalability. The submission VM dispatched a series of workflow runs to the deployment VM, with the number of runs ranging from 2 to 8192 (i.e., 2, 4, 8, …, 4096, 8192). Each run triggered the execution of a minimal Snakemake workflow on the WESkit worker comprising a single task that induced a 10-second wait, serving as a controlled workload for performance evaluation. To prevent interference from concurrent submissions, all job submissions were performed sequentially. Total runtime was measured from the initiation of the first submission to the completion of the final run. The benchmark results, presented in Figure S2, demonstrate the scalability of WESkit under increasing workload conditions. Find the benchmark data in Table S1. The reported metrics include the

- Submission time: The time elapsed from sending the first run request to receiving the last HTTP response from the server,
- Total time: The time elapsed from the start of the submission to the completion of execution,

reported for each batch. Both submission and initialization times increase proportionally with the number of concurrently submitted jobs, while the per-job runtime remains stable or exhibits a slight decrease for larger batches.

The benchmark design follows our currently largest use-case for WESkit at the DKFZ, depicted in Figure S3, which requires an average submission of 1800 samples per day. However, the data doesn’t come in uniform rates but often in clusters, therefore we assume temporarily higher rates of run submissions. Table S1 shows that in our benchmarks WESkit was able to handle 2000 submissions in under 2 minutes, exceeding the requirements of this use case.

| **Workers** | **Jobs** | **Submission Time (s)** | **Submission T.**  **per Run (s)** | **Total Time (s)** | **Total T.**  **per Run (s)** |
| --- | --- | --- | --- | --- | --- |
| 1 | 1 | 0.27 | 0.27 | 11.84 | 11.84 |
|  | 2 | 0.29 | 0.15 | 11.68 | 5.84 |
|  | 4 | 0.24 | 0.06 | 11.65 | 2.91 |
|  | 8 | 0.43 | 0.05 | 11.84 | 1.48 |
|  | 16 | 0.93 | 0.06 | 12.34 | 0.77 |
|  | 32 | 1.81 | 0.06 | 22.53 | 0.70 |
|  | 64 | 3.54 | 0.06 | 43.90 | 0.69 |
|  | 128 | 6.9 | 0.05 | 76.18 | 0.60 |
|  | 256 | 13.47 | 0.05 | 141.64 | 0.55 |
|  | 512 | 27.3 | 0.05 | 281.52 | 0.55 |
|  | 1024 | 54.32 | 0.05 | 559.89 | 0.55 |
|  | 2048 | 108.19 | 0.05 | 1109.02 | 0.54 |
|  | 4096 | 215.74 | 0.05 | 2207.93 | 0.54 |
|  | 8192 | 433.32 | 0.05 | 4414.80 | 0.54 |
| 2 | 1 | 0.28 | 0.28 | 11.87 | 11.87 |
|  | 2 | 0.11 | 0.06 | 11.67 | 5.84 |
|  | 4 | 0.21 | 0.05 | 11.61 | 2.90 |
|  | 8 | 0.43 | 0.05 | 11.82 | 1.48 |
|  | 16 | 0.9 | 0.06 | 12.29 | 0.77 |
|  | 32 | 2.02 | 0.06 | 13.42 | 0.42 |
|  | 64 | 3.58 | 0.06 | 23.25 | 0.36 |
|  | 128 | 6.99 | 0.05 | 44.27 | 0.35 |
|  | 256 | 14.1 | 0.06 | 77.17 | 0.30 |
|  | 512 | 27.97 | 0.05 | 142.83 | 0.28 |
|  | 1024 | 55.66 | 0.05 | 282.33 | 0.28 |
|  | 2048 | 111.98 | 0.05 | 560.95 | 0.27 |
|  | 4096 | 223.09 | 0.05 | 1110.80 | 0.27 |
|  | 8192 | 446.97 | 0.05 | 2215.53 | 0.27 |
| 5 | 1 | 0.31 | 0.31 | 12.07 | 12.07 |
|  | 2 | 0.15 | 0.08 | 11.57 | 5.79 |
|  | 4 | 0.25 | 0.06 | 11.66 | 2.92 |
|  | 8 | 0.44 | 0.06 | 11.85 | 1.48 |
|  | 16 | 0.88 | 0.06 | 12.29 | 0.77 |
|  | 32 | 1.76 | 0.06 | 13.18 | 0.41 |
|  | 64 | 4.01 | 0.06 | 15.43 | 0.24 |
|  | 128 | 7.29 | 0.06 | 23.87 | 0.19 |
|  | 256 | 14.45 | 0.06 | 36.23 | 0.14 |
|  | 512 | 29.34 | 0.06 | 65.63 | 0.13 |
|  | 1024 | 58.57 | 0.06 | 120.74 | 0.12 |
|  | 2048 | 117.56 | 0.06 | 230.55 | 0.11 |
|  | 4096 | 247.04 | 0.06 | 461.85 | 0.11 |
|  | 8192 | 471.18 | 0.06 | 891.32 | 0.11 |
| 10 | 1 | 0.27 | 0.27 | 11.98 | 11.98 |
|  | 2 | 0.13 | 0.07 | 11.53 | 5.77 |
|  | 4 | 0.24 | 0.06 | 11.65 | 2.91 |
|  | 8 | 0.43 | 0.05 | 11.83 | 1.48 |
|  | 16 | 0.86 | 0.05 | 12.26 | 0.77 |
|  | 32 | 1.82 | 0.06 | 13.23 | 0.41 |
|  | 64 | 3.64 | 0.06 | 15.06 | 0.24 |
|  | 128 | 7.29 | 0.06 | 18.70 | 0.15 |
|  | 256 | 14.59 | 0.06 | 26.02 | 0.10 |
|  | 512 | 30.59 | 0.06 | 42.01 | 0.08 |
|  | 1024 | 62.26 | 0.06 | 73.70 | 0.07 |
|  | 2048 | 126.82 | 0.06 | 138.25 | 0.07 |
|  | 4096 | 256.08 | 0.06 | 267.49 | 0.07 |
|  | 8192 | 515.27 | 0.06 | 526.69 | 0.06 |

**Table S1: Benchmark Results.** The benchmark data is organized into four sections corresponding to deployments with 1, 2, 5, and 10 workers, respectively. For each deployment configuration, batches ranging from 1 to 8192 jobs were submitted and executed.

# Workflow execution services

| Feature | WESkit | Sapporo | WfExS |
| --- | --- | --- | --- |
| Workflow-systems | Snakemake, Nextflow, extensions possible | no restriction; adaptation of run.sh script required | Nextflow and CWL |
| Technologies | FLASK app, celery, mongodb | FastAPI | python, crypt4gh, ro-crate |
| WES version | WES 1.1.0 | WES 1.1.0 | not supported |
| cli-client | yes | no | just cli |
| gui-client | yes | yes | no |
| license | MIT | Apache2.0 | Apache2.0 |
| deployment | docker, k8s | docker | docker, podman, apptainer |
| HPC/HTC support | native via ssh | possible through adaptation of run.sh; co-location with cluster required |  |
| Focus | HPC support, monitoring, configuration; stable execution of managed workflows for scientists; a simple tool to manage a small number of workflows and make them accessible to researchers. | Sapporo’s aim is to provide unified implementation for different workflow systems, thereby giving scientists easier access to various workflow systems. | Reproducible execution of workflows in an isolated environment. (https://f1000research.com/posters/13-550) |

**Table S2: WES Implementations**. Compares three available workflow execution systems regarding the underlying system, deployment strategy, compatibility with GA4GH standard, and focus.

# User Tutorial

This is a step-by-step guide to deploy WESkit locally. After deploying, we will show how to submit a test job and monitor its progress. If you have not used WESkit yet, this tutorial will take approximately 15-20 minutes. Make sure your system fulfills the following prerequisites.

**Requirements**

- Linux/Unix
- Docker
- Python, Pip

**STEP 1: Deploy WESkit**

WESkit is deployed locally using the deployment repository ([One Touch Pipeline / WESkit / deployment · GitLab](https://gitlab.com/one-touch-pipeline/weskit/deployment)). After finishing STEP 1, an instance of WESkit will be running in Docker on your local machine. Clone the WESkit Deployment repository:

$ git clone https://gitlab.com/one-touch-pipeline/weskit/deployment

$ cd deployment

Generate self-signed certificates:

$ ./generateDevelopmentCerts.sh

Deploy WESkit in the background using *docker compose*:

$ docker compose up -d

At this point you should have several containers in the background running WESkit. By default, WESkit will listen on localhost. It exposes all endpoints defined in the GA4GH WES API.

**STEP 2: Install weskit-client**

Having WESkit deployed allows us to test the deployment. In STEP 2, the *weskit-client* module will be installed and used to test the deployment. Install the *weskit-client* module using pip:

$ pip install weskit-client

To use the local WESkit instance, the client must be connected to the WESkit API via an environment variable. Here we assume that the service is available at *localhost*. Set the environment variable:

$ export WESKIT_API_URL="https://localhost/"

Validate the connection to the WESkit instance by requesting the *service-info* API endpoint.

$ weskit-client service-info

**STEP 3: Submit a Job**

Finally, weskit-client will be used to submit a job to the WESkit instance. In STEP 3, a test job will be submitted and monitored. The basic installation of WESkit contains two demo workflows (*wf1* and *wf2*). Using weskit-client, all workflows available on the WESkit instance can be displayed. List all available workflows:

$ weskit-client workflows

+--------+--------+-----------+--------------------+---------------------------------------------------+

| Name | Type | Version | URI | Description |

+========+========+===========+====================+===================================================+

| wf1 | SMK | 7.32.3 | file:wf1/Snakefile | This document describes the configuration for WF1 |

+--------+--------+-----------+--------------------+---------------------------------------------------+

| wf2 | SMK | 7.32.3 | file:wf2/Snakefile | This document describes the configuration for WF2 |

+--------+--------+-----------+--------------------+---------------------------------------------------+

To submit a workflow, a config file must be provided. weskit-client can be used to generate an empty template config and then the user has to provide required workflow parameters manually. Initialize *wf1* and create the config file template:

$ weskit-client init --name wf1

Workflow 'wf1' initialized and '20250630_config_wf1.yaml' created.

You will find a file '<date>_config_wf1.yaml' created in the current working directory. This file contains the parameters required by the requested workflow. At this point the user has to fill in all parameters before submitting the workflow. Open the file and fill in the text field, e.g.:

text: 'put any text here'

Save the config file. Now the workflow can be executed by providing *name* and *config* to weskit-client. Start the execution of *wf1*:

$ weskit-client exec --name wf1 --config 20250630_config_wf1.yaml
Executed workflow 'wf1' with config '20250630_config_wf1.yaml'. Run-id: f3c43665-7d1d-412d-8a59-99fc9e35bd67

You can get an overview of your jobs using weskit-client. This helps to monitor running and completed jobs. List all runs:

$ weskit-client runs

+--------------------------------------+---------+--------------------+--------------+
| ID | State | workflow | start_time |
+======================================+=========+====================+==============+
| f3c43665-7d1d-412d-8a59-99fc9e35bd67 | RUNNING | file:wf1/Snakefile | |
+--------------------------------------+---------+--------------------+--------------+

WESkit reports details for a specific run, if a *run id* is provided. You can find the run id in the run overview above. Inspect the results of a single run using the --*rid* option:

$ weskit-client runs --rid f3c43665-7d1d-412d-8a59-99fc9e35bd67

{'identity': 'not-logged-in-subject',

'outputs': {'filesystem': ['hello_world.txt',

'config.yaml',

'.snakemake/log/2025-07-03T061317.935171.snakemake.log',

'.snakemake/metadata/aGVsbG9fd29ybGQudHh0']},

…

'state': 'COMPLETE'}

This shows additional information about the run like state, log, outputs etc.

# Supplementary Figures

Figure S1: WESkit Architecture


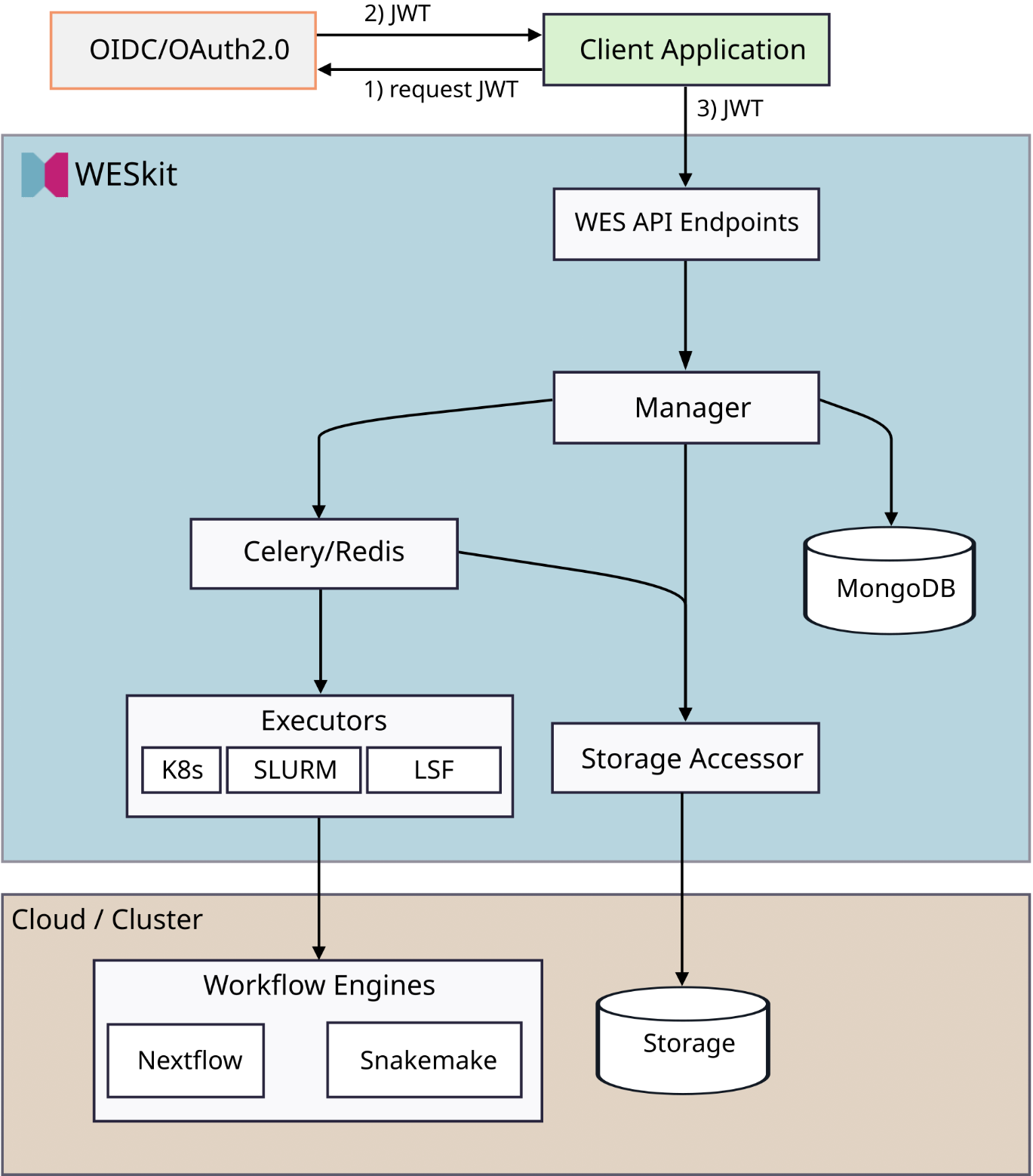


**Figure S1: WESkit System Architecture.** The figure illustrates the high-level architecture of the WESkit System and its interactions with external components. Arrows between components represent data flow.
Communication with WESkit begins when the client application requests a JWT (JSON Web Token) from an OIDC/OAuth2.0 provider. The JWT is returned to the client and used to authenticate with the WESkit system. After successful authentication, a workflow execution request is sent to the WES API Endpoints which handle incoming requests. Guided by the Manager, it transforms the request into a workflow run and wraps it into a submission command based on the configured workflow engine (Nextflow or Snakemake) and the executor (Kubernetes (K8s), SLURM, LSF).
The Manager coordinates workflow submission by forwarding the tasks to Celery for scheduling on the cluster or cloud. It also communicates with MongoDB to store and retrieve execution metadata. The Storage Accessor is responsible for setting up the execution environment and managing attachment transfer.

Figure S2: Benchmark
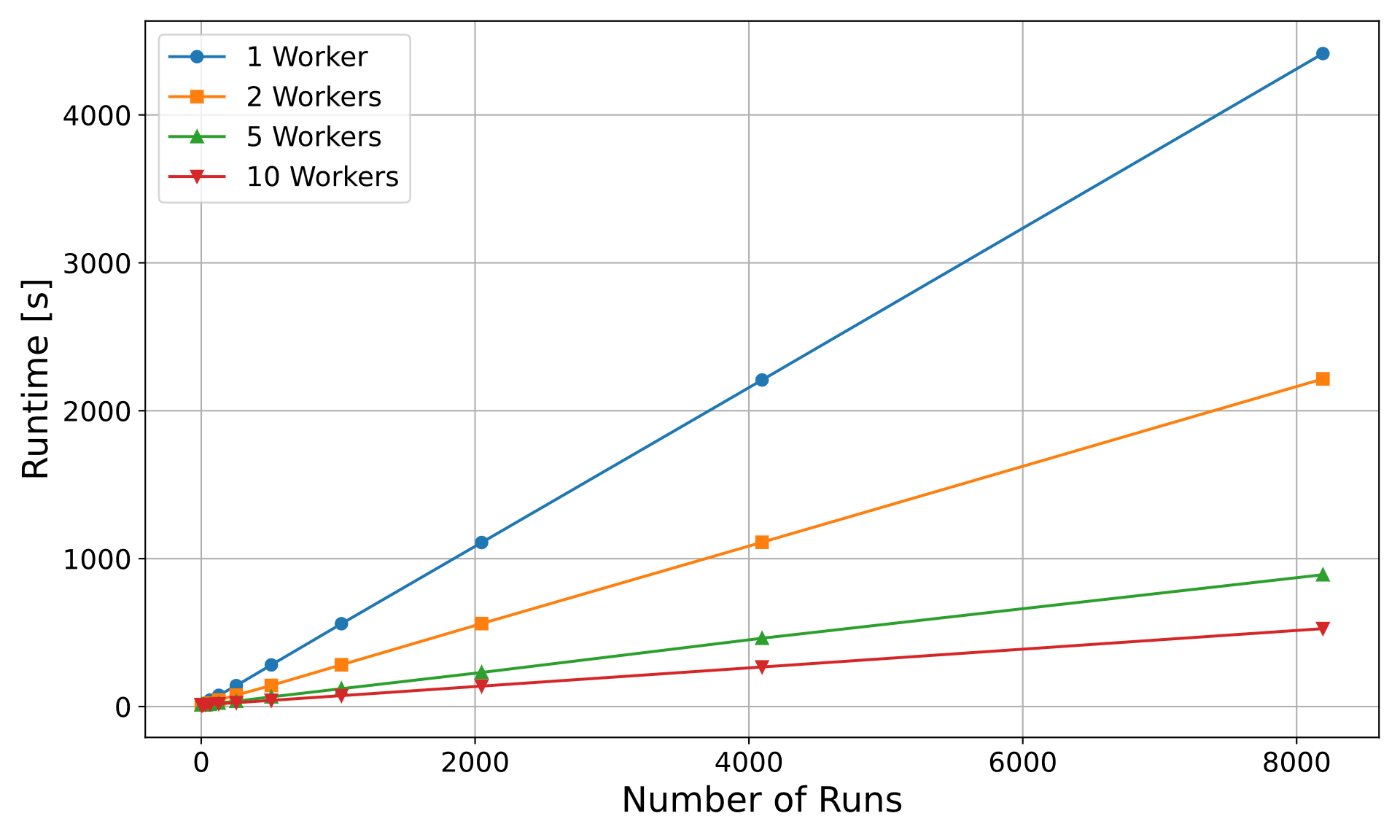


**Figure S2: Scalability and Runtime Reduction.** Runtime for varying numbers of workflow submissions (runs) scales with the numbers of workers.

Figure S3: OTP-WESkit use-case


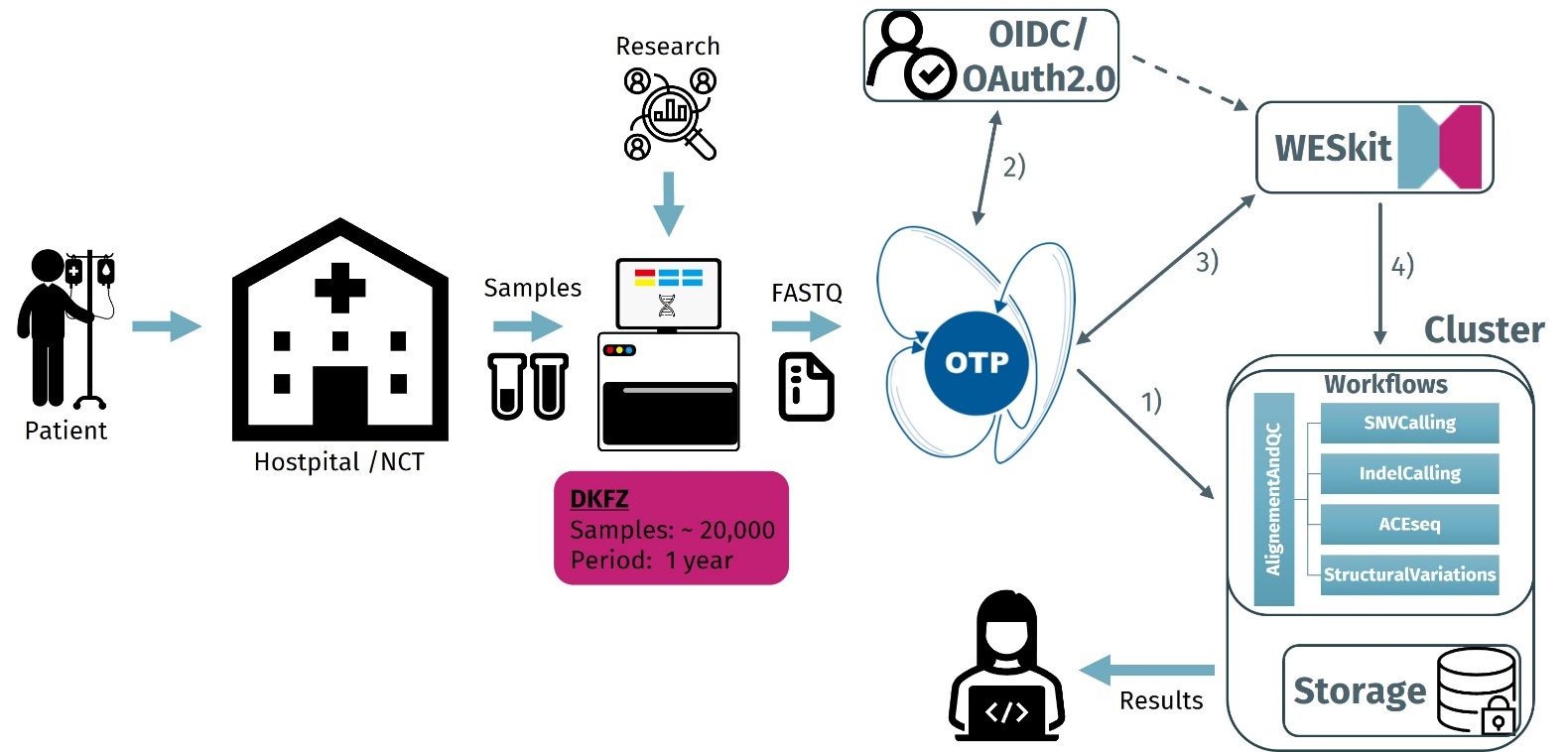


**Figure S3: Analysis pipeline.** This figure illustrates sequencing procedure of a cancer sample and the anticipated integration of WESkit. It starts with patients being diagnosed with cancer at the hospital or the “Nationales Center für Tumorerkrankungen (NCT)”. Extracted samples are being sent to DKFZ for sequencing and further processing. Additionally, research groups may provide their samples for sequencing. The sequencing results (FASTQ files) are sent for downstream processing via One-Touch-Pipeline (OTP) to WESkit that triggers the execution. In more detail, OTP 1) prepares the project and run directory, 2) an access token for WESkit authentication is obtained from OIDC identity provider, 3) the execution request is sent to WESkit, and 4) initiates workflow execution on the computing cluster. Hereby WESkit and OTP communicate the status of the job states. Once the analysis is done, bioinformatician may proceed with downstream analysis and data interpretation. The pipeline presents an anticipated sequencing of 20,000 samples per year based on the data throughput of the last 12 months. As each sample requires quality filtering and alignment before the final analysis, WESkit is expected to initiate 460,000 runs per year for this use case.
